# Obesity-Induced PVAT Dysfunction and Atherosclerosis Development: The Role of GHSR-1a in Increased Macrophage Infiltration and Adipocytokine Secretion

**DOI:** 10.3390/jcdd12030087

**Published:** 2025-02-26

**Authors:** Sorin Nicolae Peiu, Florin Zugun-Eloae, Bogdan Stoica, Ecaterina Anisie, Diana Gabriela Iosep, Mihai Danciu, Iustina Silivestru-Crețu, Fawzy Akad, Andrei Nicolae Avadanei, Laura Condur, Radu Florin Popa, Veronica Mocanu

**Affiliations:** 1 Department of Vascular Surgery, “Grigore T. Popa” University of Medicine and Pharmacy, 16, Universitatii Street, 700115 Iasi, Romania; sorin-nicolae.peiu@umfiasi.ro (S.N.P.); andrei.avadanei@umfiasi.ro (A.N.A.); radu.popa@umfiasi.ro (R.F.P.); 2 Department of Morpho-Functional Sciences II (Pathophysiology), “Grigore T. Popa” University of Medicine and Pharmacy, 16, Universitatii Street, 700115 Iasi, Romania; iustina-silvia.gs.silivestru@d.umfiasi.ro (I.S.-C.); fawzy_akad@umfiasi.ro (F.A.); 3 Department of Morpho-Functional Sciences I (Immunology), “Grigore T. Popa” University of Medicine and Pharmacy, 16, Universitatii Street, 700115 Iasi, Romania; 4 Regional Institute of Oncology, TRANSCEND Research Centre, 2-4, General Mathias Berthelot, 700483 Iasi, Romania; catianisie@yahoo.com; 5 Department of Morpho-Functional Sciences II (Biochemistry), “Grigore T. Popa” University of Medicine and Pharmacy, 16, Universitatii Street, 700115 Iasi, Romania; bogdan.stoica@umfiasi.ro; 6 Department of Morpho-Functional Sciences I (Morphopathology), “Grigore T. Popa” University of Medicine and Pharmacy, 16, Universitatii Street, 700115 Iasi, Romania; diana.iosep@umfiasi.ro (D.G.I.); mihai.danciu@umfiasi.ro (M.D.); 7 Department of Morpho-Functional Sciences I (Anatomy), “Grigore T. Popa” University of Medicine and Pharmacy, 16, Universitatii Street, 700115 Iasi, Romania; 8 Department of Family Medicine, Faculty of Medicine, Ovidius University, 124, Bd. Mamaia, 900527 Constanta, Romania; lauracondur@yahoo.com

**Keywords:** ghrelin, GHSR-1a, atherosclerotic plaque, perivascular adipose tissue (PVAT), inflammation, macrophage, peripheral artery disease (PAD)

## Abstract

In obesity, recent research revealed that increased expression of the growth hormone secretagogue receptor (GHSR) in macrophages plays a pivotal role in the development of meta-inflammation, promoting macrophage infiltration and pro-inflammatory polarization. This study aimed to examine the association between GHSR-1a expression in atherosclerotic plaques and adjacent perivascular adipose tissue (PVAT) from 11 patients with obesity and peripheral artery disease (PAD) who underwent revascularization procedures. Immunohistochemistry was used to assess the expression of CD68, CD80, and CD14, while tissue homogenate levels of adiponectin, leptin, IL-6, and CRP were quantified via ELISA. Serum markers of inflammation were also measured. Among patients with GHSR-1a-positive (+) macrophages in atherosclerotic plaques, we observed significantly higher white blood cell counts and platelet-to-lymphocyte ratios in serum, a lower adiponectin-to-leptin ratio, and elevated IL-6 levels in both arterial and PVAT homogenates. Our findings suggest a link between GHSR-1a and macrophage/monocyte infiltration, macrophage polarization, and adipocytokine secretion in atherosclerotic plaques associated with obesity-induced PVAT dysfunction.

## 1. Introduction

Ghrelin is the endogenous ligand of the growth hormone secretagogue receptor (GHSR-1a), a G protein-coupled receptor [1]. Desacyl-ghrelin (DAG), the most abundant form of plasma ghrelin, lacks an octanoyl moiety, leading to decreased GHSR-1a affinity, which means that only micromolar concentrations of DAG initiate receptor signaling, whereas acylated ghrelin (AG) activates GHSR-1a in the nanomolar concentration range [2,3] by acylation of the third serine through ghrelin O-acyltransferase (GOAT). GHSR-1a and its truncated form, GHSR-1b, are encoded by the same gene and differentiated by the number of transmembrane domains and roles, GHSR-1b being widely considered nonfunctional due to its inability to bind to ghrelin [4], despite having some influence on ghrelin-induced GHSR-1a-mediated signaling [5]. GHSR-1a modulates metabolic homeostasis during energy imbalance, cardiovascular functions, gastric acid secretion, and immune responses. Several loss-of-function mutations of GHSR-1a, such as Ala204Glu and Phe279Leu, lead to obesity in humans, caused by a compensatory response in the form of upregulated expression of GHSR-1a [6].

GHSR-1a is mainly expressed in the pituitary gland and the brain, but also in peripheral tissues. The ghrelin receptor GHSR-1a has increased density in carotid arteries or saphenous vein grafts affected by atherosclerosis [7]. GHSR-1a is highly expressed in macrophages and has a cell-autonomous effect in macrophages, being involved in the polarization and immune remodeling of macrophages [8].

In more studies, the ghrelin–GHSR-1a axis exerts anti-inflammatory effects by suppressing the pro-inflammatory cytokine secretion and reducing the inflammation. The anti-inflammatory effects of GHSR-1a are facilitated by GHSR-1a signaling mediated by ghrelin. GHSR-1a was reported to be expressed in human monocytes, and ghrelin administration reduces the secretion of pro-inflammatory cytokines (interleukin [IL]-1b and IL-6) in lipopolysaccharide-treated monocytes [8]. Also, ghrelin administered subcutaneously by micro-osmotic pumps blocked the progression of nonalcoholic steatohepatitis by GHSR-1a-mediated inhibition of M1 pro-inflammatory macrophage polarization in the hepatic sinusoids of HFD mice [9].

In the cardiovascular system, ghrelin has anti-inflammatory effects, inhibits atherosclerotic plaque formation, and promotes plaque stability [10]. Ghrelin has a direct interplay with its receptor GHSR-1a in the initiation and development of atherosclerosis by increasing nitric oxide availability and improving the endothelial function, reducing oxidative stress and inflammation, and improving insulin sensitivity and lipotoxicity [11,12,13]. Also, the aorta of GHSR-1a knockout mice had increased atherosclerotic plaque instability characterized by highly expressed pro-inflammatory vascular cell adhesion protein 1 (VCAM-1), intercellular adhesion molecule 1 (ICAM-1), CD4+ T cells, and MAC-3+ macrophages [10]. These studies suggest the anti-atherosclerotic effects of the ghrelin axis in the experimental models of atherosclerosis.

By contrast, other studies indicated that GHSR-1a signaling in macrophages plays a pivotal role in the increased inflammation in specific conditions. GHSR-1a deficiency leads to macrophage dysfunction in response to LPS stimulation and reduced lung inflammation [14]. Similar pro-inflammatory involvement of GHSR-1a was observed in experimental colitis, where the lack of GHSR significantly attenuated colitis at both clinical and pathological levels, reducing colonic pro-inflammatory cytokines [15]. In adipose tissue, the expression of GHSR-1a increased during aging in mice, and the ablation of GHSR-1a attenuates the age-associated increase in pro-inflammatory peritoneal macrophages and reduces inflammation in visceral WAT [16]. In high-fructose corn syrup (HFCS)-fed mice, deleting GHSR reduces pro-inflammatory macrophage infiltration into adipose tissue, ameliorating adipose inflammation and whole-body insulin sensitivity [17].

In a previous paper, we proposed that ghrelin may influence plaque stability and vascular inflammation. We examined ghrelin’s impact on vascular health, particularly in the context of metabolic syndrome and age-related vascular alterations. Ghrelin expression was higher in complicated plaques as compared to uncomplicated plaques of PAD patients. Ghrelin expression was higher in perivascular tissue adjacent to complicated plaques as compared to tissues around uncomplicated plaques, suggesting a potential role for ghrelin in inflammatory modulation during atherosclerosis progression, particularly in complicated plaques [18].

Data on GHSR-1a’s role in arterial disease are scarce. GHSR-1a has been previously shown to promote neointimal formation in a mouse model of femoral artery wire injury by proliferation and migration of smooth muscle cells via Akt and ERK1/2 pathways [19]. GHSR-1a mediates the stimulation of nitric oxide production by ghrelin in endothelial cells of the human aorta [20]. However, coronary atherosclerosis was the focus of research, and GHSR-1a was found to mediate cardiac remodeling after myocardial infarction [21,22]. Ghrelin has been investigated for its role in angiogenesis and improved recovery from ischemia as a potential treatment for PAD [23], but the role of GHSR-1a in inflammation, a major risk factor for PAD progression, has not yet been established.

The present study aims to investigate the association of GHSR-1a immunopositive (+) cells from atherosclerotic plaque and PVAT with inflammatory markers (tissue concentrations of CRP, Il-6, leptin, and adiponectin) by studying the arteries harvested from patients with obesity and PAD.

## 2. Materials and Methods

### 2.1. Study Participants

The study group was recruited from PAD patients admitted to the Vascular Surgery Service of “St. Spiridon” County Emergency Hospital, Iasi (Romania). Inclusion criteria were patients over the age of 50 years old, moderate to severe symptomatic PAD with ischemic rest pain or ulceration and gangrene present for at least 2 weeks, and patients undergoing open surgical or endovascular revascularization for PAD. Exclusion criteria were asymptomatic PAD, mild claudication or no functional impairment, and patients with dementia who could not provide informed consent.

The 11 patients were predominantly men (10 male, 90.9%, and 1 female, 9.1%) with a mean age of 66.64 (±8.053) years, ranging from 53 to 86 years old (3 men aged 50–59 y., 27.3%; 4 men and 1 woman aged 60–69 years., 45.5%; 2 men aged 70–79 years., 18.2%; 1 man aged > 80 years, 9.1%). All patients had obesity (BMI > 30 kg/m^2^, ranging from 30.32 to 39.67 kg/m^2^) and hypertension. Other comorbidities included type II diabetes mellitus (45.45%) and dyslipidemia (81.82%). Most patients were smokers (72.73%). All patients received similar medications, including antihypertensives, cholesterol-lowering drugs, and platelet antiaggregants.

Body mass index (BMI) was calculated as a ratio of weight and height. The central obesity index was determined as the waist circumference to height ratio.

### 2.2. Sample Collection

Tissues were obtained after the following revascularization procedures: distal femoropopliteal bypass with reversed saphenous vein, femoropopliteal bypass with reinforced PTFE (polytetrafluoroethylene) synthetic graft, aorta–bifemoral bypass with silver-coated Dacron synthetic graft, and lower limb amputations below and above the knee. Tissue samples were obtained from the superficial femoral artery, popliteal artery, anterior tibial artery, and external iliac artery.

Arterial and perivascular adipose tissues were postoperatively harvested. For optimal clinical relevance, areas with advanced atherosclerosis were selected based on angiographic images obtained during atherectomy.

The tissue samples varied in length from 0.5 cm to 18 cm. These tissues were routinely processed by fixation in 10% buffered formalin (for 12–20 h depending on the sample’s size), paraffin embedding, sectioned at 4 μm, and stained with hematoxylin–eosin for optical microscope analysis.

### 2.3. Immunohistochemical Study

Detection by immunohistochemistry was performed for CD68—general macrophage marker, CD80—M1 macrophage marker, CD14—monocyte marker, and growth hormone secretagogue receptor (GHSR-1a) for which acyl ghrelin (AG) is an endogenous ligand, DAG being GHSR-1a independent. Their expression was assessed in the atherosclerotic arterial tissue and the PVAT samples obtained from obese PAD patients.

The immunohistochemical study used monoclonal antibodies: anti-CD68 (rabbit anti-human, dilution 1:100, clone 514H12, Novocastra, Newcastle, UK), anti-CD80 (rabbit anti-human, dilution 1:100, clone EPR1157, Abcam, Cambridge, UK), anti-CD14 (rabbit anti-human, dilution 1:50, clone EPR3653, Abcam, Cambridge, UK), and anti-ghrelin (rabbit anti-human, dilution 1:50, clone EPR20502, Abcam, Cambridge, UK). A specific epitope retrieval solution with a pH of 9 was used at 96 °C for 25 min as a pretreatment. UltraVision LP Detection System and 3,3′-Diaminobenzidine (DAB) chromogen (ThermoFisher Scientific, Fremont, CA, USA) were used for the detection of immunoreactive signals.

For the GHSR-1a assessment, a semi-quantitative score was obtained from the sum of the intensity and proportion of positive cells. The intensity of the GHSR-1a immunolabeling was scored as 0 = negative, 1 = weak, 2 = moderate, and 3 = strong. We determined the graphical distribution of the score in arterial tissues and PVAT by calculating the receiver operating characteristic (ROC) curve and the area under the curve (AUC). The AUC was 0.867 in arterial tissues, with a cut-off point of 1, and the AUC was 0.654 in PVAT, with a cut-off point of 1. Two groups were formed: 0 and 1 were named GHSR-1a (−), and 2–6 were named GHSR-1a (+).

### 2.4. Biochemical Analysis

The quantification of adiponectin, leptin, IL-6, and CRP in tissue homogenates was performed with ELISA. The serum markers of inflammation were provided by preoperative laboratory testing of the patients enrolled in our study.

The commercially available Human CRP (C-reactive protein) Sandwich ELISA Kit, Catalog No: E-EL-H0043 (Elabscience Biotechnology Co., Ltd., Houston, TX, USA) with a sensitivity of 0.23 ng/mL and a detection range of 0.39–25 ng/mL was used for inflammation measurement according to the manufacturer’s instructions. Adiponectin concentrations were analyzed using the Fine Test Human ADP (Adiponectin) ELISA Kit, Catalog No: EH2593 (Fine Test Biotech Co., Ltd., Wuhan, China) with a sensitivity of 0.938 ng/mL and a detection range of 1.563–100 ng/mL following the manufacturer’s protocol. IL-6 levels were measured with the Human IL-6 (Interleukin 6) ELISA Kit, Catalog No: E-EL-H0102 (Elabscience Biotechnology Co., Ltd., Houston, TX, USA), with a sensitivity of 4.69 pg/mL and a detection range of 7.81–500 pg/mL. Leptin concentrations were measured with the Human LEP (Leptin) ELISA Kit, Catalog No: EH0216 (Fine Test Biotech Co., Ltd., Wuhan, China), with a sensitivity of 18.75 pg/mL and a detection range of 31.25–2000 pg/mL. Concentrations were calculated based on standard curves.

### 2.5. Statistical Analysis

Data were expressed as mean ± SD. Data were statistically analyzed using IBM Statistical Package for the Social Sciences (SPSS) version 19. Differences between GHSR-1a immunonegative and GHSR-1a immunonegative atherosclerotic plaque were analyzed for statistical difference using the non-parametric Mann–Whitney test. A *p*-value of <0.05 was considered statistically significant. Due to the small number of participants and the heterogeneity among tissues, a 95% confidence interval (CI) was calculated for the significant variables (*p* < 0.05).

## 3. Results

Patients were divided into two study groups: GHSR-1a immunonegative atherosclerotic plaque, GHSR-1a (−), and GHSR-1a immunopositive atherosclerotic plaque, GHSR-1a (+). Patients from these groups had similar ages, body mass index, blood glucose, total cholesterol, and total cholesterol/HDL-cholesterol ratio. GHSR-1a (+) patients had an increased serum CRP, fibrinogen, and platelet-to-lymphocyte ratio compared to GHSR-1a (−) patients. Differences in the central obesity index, white blood cell count, platelet-to-lymphocyte ratio, and triglycerides between GHSR-1a (+) and GHSR-1a (−) patients suggest that GHSR-1a expression influences the accumulation of abdominal fat, the immune response, inflammation, and lipid metabolism, thereby potentially altering cardiovascular risk profiles. The demographic and metabolic features of study participants are shown in Table 1.

In arterial tissue, the concentrations of the ADP/LEP ratio and IL-6 were significantly higher in the GHSR-1a (+) group as compared to the GHSR-1a (+) group. Except for adiponectin, which was present in only slightly higher levels in GHSR-1a (−) and (+) patients and in arterial tissues and PVATs, leptin and CRP levels were increased in the arterial tissues and PVATs of GHSR-1a (+) patients as compared with the GHSR-1a (−) patients. Table 2 shows the tissue concentrations of inflammatory markers measured by ELISA in arterial and PVAT homogenates.

**Figure 1:**
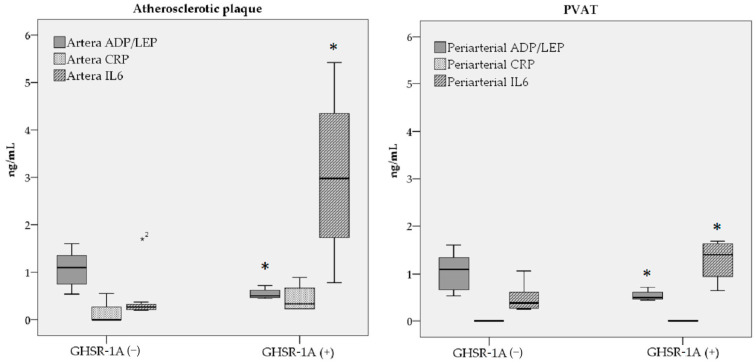
Adiponectin-to-leptin (ADP/LEP) ratio, CRP, and IL-6 levels in the atherosclerotic plaque and PVAT of GHSR-1a (−) and GHSR-1a (+) PAD patients. * a *p*-value <0.05. Boxplots detected one outlier for the IL-6 measured in the atherosclerotic plaque in the group GHSR-1a (−).

These results suggest varying degrees of inflammation, metabolic function, and immune response in GHSR-1a (−) and (+) patients, indicating that GHSR-1a status might influence the levels of adiponectin, leptin, CRP, and IL-6. An increase in IL-6 levels was found in GHSR-1a (+) patients in both arterial tissues and PVATs, as shown in Figure 1.

Arterial complications such as calcification, ulceration, and thrombosis were more prevalent in GHSR-1a (+) patients compared to GHSR-1a (−) patients, suggesting that GHSR-1a expression may be associated with a higher risk of developing arterial complications. The macrophage immunohistochemistry study showed higher levels of inflammation in GHSR-1a (+) patients than in GHSR-1a (−) patients. All GHSR-1a (+) patients showed CD68 positivity, whereas in GHSR-1a (−) patients, 42.9% had positive CD68 expression. A total of 75% of GHSR-1a (+) patients showed high CD80 expression, while 71.4% of GHSR-1a (−) patients had no CD80 expression. All GHSR-1a (−) patients showed no CD14 expression, whereas only 50% of GHSR-1a (+) patients had no CD14 expression. Markers on the surface of immunohistochemically found macrophages in the arterial tissue and PVAT of GHSR-1a (−) and (+) PAD patients are shown in Table 3.

**Figure 2:**
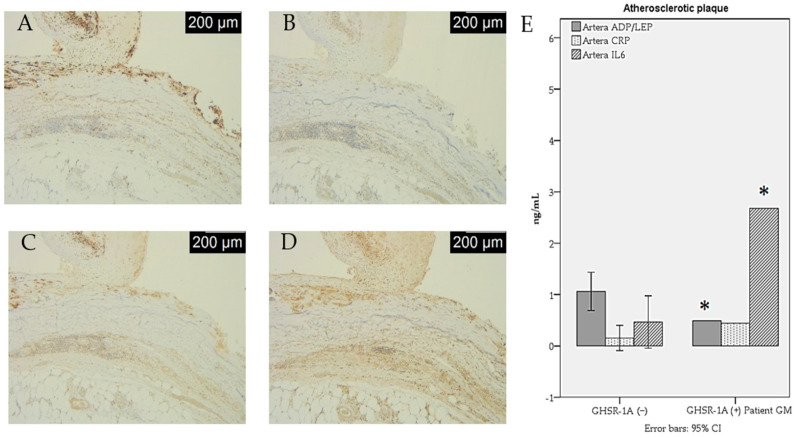
Macrophage infiltration and adipocytokine secretion in the atherosclerotic (ATS) plaque of a GHSR-1a (+) PAD patient. (**A**) GHSR-1a-positive in the complicated plaque (IHC, anti-GHSR-1a antibody, ×25). (**B**) CD68 immunoexpression in the complicated plaque (IHC, anti-CD68 antibody, ×25); (**C**) CD80 immunoexpression in the complicated plaque (IHC, anti-CD80 antibody, ×25); (**D**) CD14 immunoexpression in the complicated plaque (IHC, anti-CD14 antibody, ×25); (**E**) adipocytokine secretion in the complicated plaque (ELISA measurements in tissue homogenate). * a *p*-value < 0.05.

**Figure 3:**
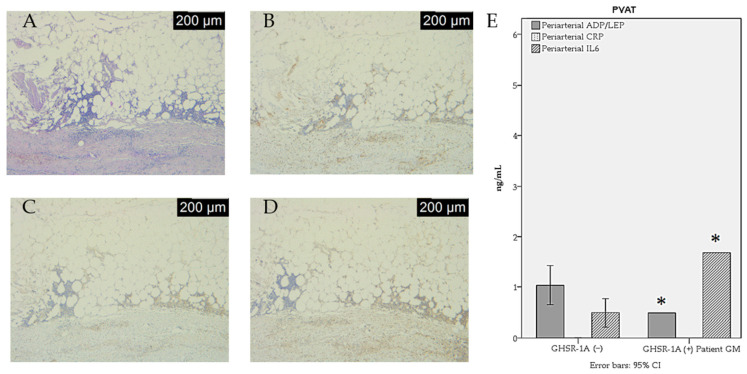
Macrophage infiltration and adipocytokine secretion in perivascular arterial adipose tissue (PVAT) of a GHSR-1a (+) PAD patient. (**A**) Inflammatory infiltration in the PVAT (hematoxylin–eosin, ×25). (**B**) CD68 immunoexpression in the PVAT (IHC, anti-CD68 antibody, ×25); (**C**) CD80 immunoexpression in the PVAT (IHC, anti-CD80 antibody, ×25); (**D**) CD14 immunoexpression in the PVAT (IHC, anti-CD14 antibody, ×25); (**E**) adipocytokine secretion in the PVAT (ELISA measurements in tissue homogenate). * a *p*-value < 0.05.

The macrophage infiltration and adipocytokine secretion in the atherosclerotic (ATS) plaque of the GHSR-1a (+) PAD patient (Patient G.M.) are shown in Figure 2 and Figure 3.

## 4. Discussion

The present study examined the association between GHSR-1a and inflammation in the arterial and PVAT tissue of obese PAD patients. The circulating markers of inflammation, CRP and IL6, may also be associated with inflammatory conditions such as arthritis or infections. Hence, we examined the markers of atherosclerotic inflammation in tissue homogenates.

GHSR-1a (+) patients had increased levels of inflammation compared with GHSR-1a (−) patients, as expressed by macrophage immunohistochemistry, ELISA markers, serum CRP, fibrinogen, and platelet-to-lymphocyte ratio. The arterial tissue and PVAT of the superficial femoral artery, popliteal artery, anterior tibial artery, and external iliac artery were correlated with metabolic syndrome indicators, such as increased waist circumference, triglyceridemia, glycemia, and atherosclerosis.

White adipocytes produce several cytokines, including adiponectin, leptin, and IL-6 [24]. Adiponectin (ADP) and leptin (LEP) play a crucial role in insulin sensitivity and lipid metabolism. IL-6 is a pro-inflammatory cytokine that is involved in the immune response and plays a role in insulin resistance. Low ADP concentrations and an altered ratio between leptin and ADP are associated with obesity, altered insulin signaling, and an inflammatory state in obese patients [25]. Reduced levels of ADP are strongly associated with cardiovascular disease in animal models, with increased expression of anti-inflammatory genes, and suppression of pro-inflammatory gene expression (TNF α and IL-6) [26].

The adipose tissue releases adipokines that have a pro-inflammatory (leptin, IL-6) or anti-inflammatory (adiponectin) role in atherosclerosis. Perivascular adiponectin concentrations were found to be significantly higher in PAD patients with wound complications following revascularization procedures [27], but low levels of adiponectin occur in most PAD patients [28]. Ghrelin suppresses the production of adiponectin in brown adipose tissue [29], but both ghrelin and adiponectin serum levels are decreased in obese individuals [30,31]. GHSR-1a increases inflammation in adipose tissues, as shown by aged or HFD GHSR-1a knockout mice being protected against inflammation of white and brown adipose tissue by decreased macrophage infiltration and macrophage polarization towards an M2 phenotype, which is associated with stable atherosclerotic plaques and asymptomatic lesions [14,32,33]. However, GHSR-1a has been shown to have protective effects on cardiovascular tissues. Deletion of GHSR-1a increased vascular inflammation and plaque instability in atherosclerosis [10] and macrophage infiltration in hearts with fibrotic areas [34] in GHSR-1a knockout mice. These contradictory effects of GHSR-1a, i.e., pro-inflammatory effects in adipose tissue and anti-inflammatory effects in arterial and cardiac tissue, have led us to study the impact of GHSR-1a expression in PVAT on atherosclerotic inflammation.

In addition to ghrelin, the adipocyte-derived cytokines leptin and adiponectin have various effects on atherosclerosis progression. Leptin aggregates monocytes, promotes foam cell formation, and accelerates the production of pro-inflammatory cytokines [35]. Conversely, adiponectin exhibits anti-atherogenic effects by inhibiting endothelial cell apoptosis and reducing the expression of vascular adhesion molecules, foam cell formation, and the proliferation of vascular smooth muscle cells [36]. Obesity is associated with increased leptin levels and decreased ghrelin and adiponectin levels [37].

The high IL-6 levels found in our study may be explained by the predominant male sex and smoking habits and by high BMI, as confirmed in the literature [38,39]. The increased production of IL-6 might also be caused by perivascular adipose tissue dysfunction characteristic of vascular diseases [40]. The high levels of leptin and the low levels of adiponectin could also have influenced the synthesis of IL-6, which is enhanced by leptin and inhibited by adiponectin [41,42]. In our study, GHSR-1a (−) PAD patients had less IL-6 in arterial tissues than GHSR-1a (+) subjects, indicating an inflammatory role of GHSR-1a.

The levels of adiponectin in the PVAT were low in all our patients, which is corroborated by another study showing decreased levels of adiponectin in the PVAT of patients with peripheral arterial occlusive disease [43]. Decreased mRNA levels of adiponectin were also found in the mesenteric adipose tissue in a murine model of type 2 diabetes mellitus using db/db mice [44]. Adiponectin release is inhibited by pro-inflammatory cytokines such as IL-6 released from PVAT [45,46], and PVAT-derived adiponectin is reduced in obesity [47]. Endovascular injury downregulates the expression of adiponectin in PVATs of mice and rats [48].

Leptin levels were increased in the atherosclerotic plaque and PVAT of GHSR-1a (+) patients. The association of high leptin with low ghrelin was proposed as a stronger marker of early atherosclerosis than ghrelin alone in patients with metabolic syndrome [49]. The increase in pro-inflammatory adipokines, such as leptin, is a characteristic of atherosclerotic PVAT [40].

Studies have demonstrated that aging diminishes ghrelin’s ability to regulate white adipose tissue (WAT) lipolysis [50]. Evidence from ghrelin knockout mouse models indicates resistance to age-related increases in fat mass and weight gain, highlighting ghrelin’s potential role in adipose tissue homeostasis. In humans, plasma levels of acylated ghrelin progressively decline with age, even in healthy individuals. This gradual reduction in ghrelin levels, associated with both aging and weight gain, may contribute to the loss of its regulatory functions, including its capacity to mitigate adipose tissue inflammation [50].

In obesity, leptin resistance and ghrelin dysregulation are associated with pro-inflammatory responses and the chronic sub-inflammatory state observed in obesity. The increased leptin resistance associated with high levels of free fatty acid and inflammatory cytokines may contribute to the reduction in lipid oxidation in insulin-sensitive organs, leading to the accumulation of lipids (lipotoxicity) and insulin resistance [51].

Endothelial cells, macrophages, lymphocytes, and adipocytes are extra-hepatic sources of CRP. CRP involvement in atherosclerosis is due to nitric oxide inhibition in cardiovascular endothelial cells. Increased circulating CRP levels are considered a predictor of major cardiovascular events in PAD [52]. Levels of CRP showed a 3-fold increase in the periaortic adipose tissue of Goto–Kakizaki diabetic rats as compared with nondiabetic Wistar rats [53]. PVAT-derived CRP has increased levels during obesity and diabetes mellitus [54] and has been shown to promote neointimal hyperplasia after endovascular injury in HFD mice [55]. CRP was absent in PVATs in the present study, independent of the expression of GHSR-1a, even after being measured by a high-sensitivity ELISA with monoclonal and polyclonal antibodies. This might mean that CRP produced by PVAT moved into the arterial tissues or that CRP is highly concentrated in arteriosclerotic plaques and their source is the peripheral blood mononuclear cells, as shown in the literature [56].

The increased number of infiltrating CD68-, CD80-positive macrophages, and CD14-positive monocytes in the GHSR-1a (+) patients that are usually associated with chronic inflammation in atherosclerosis indicated increased macrophage infiltration and M1 macrophage polarization, leading to the release of pro-inflammatory cytokines, such as IL-6 [57], which was found in abundance in our samples. Upon activation via CD14, monocytes release the same amount of IL-6 even after 4 h under metabolic restriction, such as oxygen and glucose deprivation [58].

Our study group included aged patients with obesity and atherosclerosis, which are both associated with decreased circulating levels of ghrelin [59,60]. Most patients were men (90.9%), and they had lower levels of ghrelin than women, who have higher levels of ghrelin, especially during menopause [61]. Higher concentrations of leptin and insulin in the elderly are responsible for the low sensitivity to ghrelin found in the elderly [62]. Obesity causes dysfunctional PVAT that leads to decreased adiponectin levels and increased leptin and IL-6 levels and contributes to atherosclerosis progression [63]. Moreover, aging can increase plasma ghrelin levels and GHSR expression in adipose tissue, leading to macrophage polarization toward M1 pro-inflammatory macrophages, adipose tissue inflammation, and metabolic regulation [16]. In conclusion, ghrelin signaling plays a role in age-associated obesity and insulin resistance, mediated through increased thermogenesis in brown adipose tissue (BAT) [16]. Ghrelin receptors were first detected by immunohistochemistry in human blood vessels by Kleinz et al. 2006 [64]. The sensitivity to ghrelin is mediated by GHSR-1a expression. GHSR-1a was found in the saphenous vein, left internal mammary artery, and coronary artery of coronary artery bypass graft surgery patients [64]. Positive immunoreaction for GHSR-1a was also found in patients with chronic heart failure. The increased GHSR-1a expression was proposed to serve as a compensatory mechanism in chronic heart failure due to impaired ghrelin production in a failing myocardium [65]. The increased levels of GHSR-1a in the aorta of rats during early sepsis were attributed to the effect of lipopolysaccharides [66].

Our results are contradictory to some previous animal studies. An atherosclerosis model of HFD ApoE^−/−^ mice showed both in vivo and in vitro that hexarelin, an agonist of GHSR-1a, upregulated the expression of GHSR-1a in aortae in vivo and in macrophages in vitro and had synergistic anti-atherosclerotic effects. Hexarelin and GHSR-1a reversed the upregulation of CD68 and limited the infiltration of macrophages into intima in vivo by inhibiting the activation of the LOX-1-NFκB signaling pathway [67]. Ghrelin, acting on GHSR-1a, reduced macrophage content, total cholesterol, and LDL-cholesterol in the atherosclerotic abdominal aorta plaques in an in vivo study on male New Zealand white rabbits [68]. In both experimental studies, the role of exogenously administered ghrelin was investigated. Ghrelin’s potential as a treatment for PAD in diabetic patients was suggested by a study on diabetic mice and ghrelin knockout mice showing that exogenous ghrelin contributes to vascular repair following hindlimb ischemia by improving blood flow recovery, enhancing angiogenesis, and reducing fibrosis through specific microRNAs, such as miRNA-126 and miRNA-132 [69]. GHSR-1a knockout mice showed decreased activity of AMP-activated protein kinase (AMPK), an enzyme that maintains energy balance within cells and has protective roles in atherosclerosis by inhibiting inflammation and vascular smooth muscle cell proliferation [70].

In common with our findings, other studies show either detrimental effects of GHSR-1a or lack of GHSR-1a expression in arteries. GHSR-1a deficiency had a protective effect on vascular remodeling after injury by suppressing neointimal formation, which is mediated through the inhibition of smooth muscle cell proliferation and migration via the Akt and ERK1/2 signaling pathways [19]. Acute inflammation increased GHSR-1a mRNA in human coronary artery smooth muscle cells [71]. Absent human and rat GHSR-1a expression in the aorta and the mesenteric, cerebral, and coronary arteries suggested that ghrelin causes vasodilation and lowering of blood pressure not by directly acting on arterial smooth muscle cells or endothelial cells, but rather by decreasing sympathetic nerve activity [72].

Some of the limitations of this study include the small number of participants and not being able to analyze sex differences in our sample. The use of commercially available antibodies for GHSR detection could not distinguish between GHSR-1a and GHSR-1b. The study of heterogeneous types of arteries makes it difficult to interpret the expression of ghrelin receptors. However, our study may help to understand whether the anti-inflammatory action of ghrelin in PAD mediated by GHSR-1a can be considered a compensatory immune response and how the obesity-related PVAT dysfunction and old age can reverse this anti-inflammatory effect.

## 5. Conclusions

This is the first study to demonstrate that the immunohistochemical expression of GHSR-1a in atherosclerotic arteries predisposes to increased local inflammation in obese PAD patients, as shown by higher levels of pro-inflammatory cytokines released by adipose tissue, CD68- and CD80-positive macrophages and CD14-positive monocytes from atherosclerotic plaque and PVAT. This pro-inflammatory effect of GHSR-1a may be due to the pro-inflammatory action of obesity-related dysfunctional PVAT observed in aging.

## Figures and Tables

**Table 1:** Characteristics of the GHSR-1a (−) and GHSR-1a (+) groups.

Parameters	GHSR-1a (−) Men (n = 6) Women (n = 1)	GHSR-1a (+) Men (n = 4)	*p*-Value
Age (years)	66.57 ± 8.89	66.75 ± 7.63	0.85
Body mass index (kg/m^2^)	33.43 ± 2.96	31.25 ± 0.50	0.09
Central obesity index	0.48 ± 0.06	0.56 ± 0.03	0.04 *
White blood cell count	9337.14 ± 1383.29	13,440.00 ± 5207.10	0.04 *
Platelet-to-lymphocyte ratio	1968.36 ± 968.92	3358.38 ± 672.62	0.04 *
Total cholesterol (mg/dL)	269.71 ± 71.69	242.50 ± 29.01	0.57
Triglycerides (mg/dL)	296.29 ± 63.13	366.25 ± 16.01	0.03 *
Total Chol/HDL Chol	5.45 ± 1.67	5.02 ± 0.6	0.85
Glucose (mg/dL)	114.43 ± 32.41	119.75 ± 28.19	0.45
Hb A1C (%)	6.79 ± 0.85	6.78 ± 0.56	0.70
Serum CRP (mg/L)	27.71 ± 22.32	56.50 ± 22.13	0.06
Fibrinogen (mg/dL)	305.71 ± 120.40	461.50 ± 34.58	0.06

* A *p*-value of <0.05 was considered statistically significant.

**Table 2:** Inflammatory markers were measured by ELISA in arterial tissue and PVAT homogenates in GHSR-1a (−) and GHSR-1a (+) groups.

Parameters	GHSR-1a (−) Men (n = 6) Women (n = 1)	GHSR-1a (+) Men (n = 4)	*p*-Value
In Atherosclerotic plaque	-	-	-
Adiponectin (ng/dL)	3.94 ± 2.65	4.68 ± 3.67	0.85
Leptin (ng/dL)	5.04 ± 4.48	9.44 ± 7.54	0.26
ADP/LEP ratio	1.06 ± 0.40	0.55 ± 0.12	0.02 *
Tissue CRP (ng/dL)	1.56 ± 2.65	4.47 ± 3.11	0.12
IL-6 (ng/dL)	4.65 ± 5.51	30.37 ± 19.10	0.01 *
In PVAT	-	-	-
Adiponectin (ng/dL)	1.63 ± 0.80	1.77 ± 1.03	0.64
Leptin (ng/dL)	1.91 ± 1.60	3.43 ± 2.30	0.26
ADP/LEP ratio	1.04 ± 0.42	0.55 ± 0.12	0.04 *
Tissue CRP (ng/dL)	0	0	1.00
IL-6 (ng/dL)	4.94 ± 3.02	12.87 ± 4.63	0.02 *

Abbreviations: adiponectin (ADP), leptin (LEP), perivascular arterial adipose tissue (PVAT). * A *p*-value of <0.05 was considered statistically significant.

**Table 3:** Frequency distribution of macrophage markers that are usually associated with chronic inflammation in atherosclerosis in relationship with GHSR-1a expression.

Histological Examination and IHC	GHSR-1a (−) (n = 7)			GHSR-1a (+) (n = 4)		
	0	1 *	≥2	0	1	≥2
Atherosclerotic plaque						
Inflammation intensity	5 (71.4%)	2 (28.6)	_	_	2 (50%)	2 (50%)
Calcification	4 (57.1%)	3 2.9%)	_	_	4 (100%)	_
Ulceration	5 (71.4%)	2 (28.6%)	_	_	4 (100%)	_
Thrombosis	4 (57.1%)	3 (42.9%)	_	_	4 (100%)	_
CD68	4 (57.1%)	3 (42.9%)	_	_	4 (100%)	_
CD80	5 (71.4%)	2 (28.6%)	_	_	1 (25%)	3 (75%)
CD14	7 (100%)	_	_	2 (50%)	_	2 (50%)
PVAT						
Inflammation intensity	4 (57.1%)	3 (42.9)	_	1 (25%)	2 (50%)	1 (25%)
CD68	2 (28.6%)	2 (28.6%)	3 (42.9%)	1 (25%)	1 (25%)	2 (50%)
CD80	5 (71.4%)	2 (28.6%)	_	2 (50%)	1 (25%)	1 (25%)
CD14	6 (85.7%)	_	1 (14.3%)	2 (50%)	_	2 (50%)

* In cases of arterial complications, classified as calcification, ulceration, or thrombosis, the presence of any one of these conditions was considered a complication (code 1). Abbreviation: perivascular arterial adipose tissue (PVAT).

## Data Availability

Data are unavailable due to privacy and ethical restrictions.
